# Acute Inflammatory Demyelinating Polyneuropathy With Bowel and Bladder Incontinence Following COVID-19 Infection

**DOI:** 10.7759/cureus.17896

**Published:** 2021-09-11

**Authors:** Vijay Letchuman, Kelly M Wemhoff, Gurpreet S Gandhoke

**Affiliations:** 1 Neurological Surgery, University of Missouri–Kansas City School of Medicine, Kansas City, USA; 2 Neurological Surgery, Saint Luke’s Hospital, Kansas City, USA

**Keywords:** covid-induced guillain-barré syndrome, guillain-barré syndrome, covid-19, acute inflammatory demyelinating polyradiculoneuropathy, spinal stenosis

## Abstract

Coronavirus disease 2019 (COVID-19) has led to a global pandemic with the recent demonstration of several neurological manifestations. While there are limited reports of neurologic involvement in the context of COVID-19 infection, recent evidence has established the neuroinvasive potential of the virus.

A 57-year-old man was diagnosed with COVID-19 via a polymerase chain reaction test and treated as an outpatient with a combination of prednisone and azithromycin. Nine days after his initial diagnosis, he was admitted to the intensive care unit for acute respiratory failure where he required high-flow oxygen support at a maximum of 60 L/minute. Ten days after his admission to the intensive care unit, he was discharged requiring no oxygen at rest, but 2-3 L/minute with exertion. Nine days after his discharge, he was readmitted with a six-day history of bilateral lower extremity weakness, low back pain, diminished sensation, bowel and bladder incontinence, and decreased rectal sensation and tone. Evaluation for cauda equina syndrome was unremarkable; however, cervical magnetic resonance imaging revealed severe central cervical stenosis of C3-4 and C4-5 with spinal cord flattening and intraparenchymal T2 hyperintensity. The examination was notable for muted reflexes in the bilateral lower extremities, T10 sensory level, decreased rectal tone, and ambulation with a walker. Cerebrospinal fluid analysis revealed an albuminocytologic dissociation. Treatment with intravenous dexamethasone and immunoglobulin resulted in partial motor resolution and complete resolution of his bowel and bladder incontinence within three days of treatment.

In the face of this novel global pandemic, surgeons and clinicians should carefully evaluate patients presenting with neurologic deficits and ensure a thorough examination to accurately identify the appropriate etiology for a neurologic deficit.

## Introduction

Coronavirus disease 2019 (COVID-19) is caused by the severe acute respiratory syndrome coronavirus 2 (SARS-CoV-2) virus. While primarily a respiratory infection, it has been associated with several neurologic manifestations such as headaches, confusion, dizziness, myalgia, anosmia, and ageusia [1,2]. Recently, numerous case reports in the literature have described the association between COVID-19 and Guillain-Barré syndrome (GBS), though the specific clinical pattern is still emerging [1,3-7].

GBS is an acute, generalized polyradiculoneuropathy that is often preceded by a symptomatic viral infection such as *Campylobacter jejuni*, influenza, cytomegalovirus, or Epstein-Barr virus [8]. To date, most reports that describe post-COVID GBS have been seen in patients who presented with COVID-19 symptoms requiring hospitalization with an average GBS symptom onset of one to four weeks from the start of their COVID symptomology [6,9]. Unlike the symptomatology and classical presentation of GBS following known viral illnesses, the clinical and electrodiagnostic patterns of post-COVID GBS are still unknown at this stage of the pandemic [3,10].

Furthermore, given the novelty of the SARS-CoV-2 virus, we are at a critical period in the understanding of acute and chronic neurologic complications associated with COVID-19 infection. For clinicians in practice, it is crucial to identify the etiology of an individual’s neurologic presentation from a known etiology from a potential post-COVID complication. Here, we describe a unique patient who presented with symptoms typically associated with cauda equina syndrome and rapid bowel and bladder incontinence in the background of a recent COVID-19 infection along with an incidental finding of severe cervical central cord stenosis demonstrated radiologically.

## Case presentation

First presentation

A 57-year-old male with a medical history significant for type 2 diabetes mellitus, hypertension, chronic obstructive pulmonary disease, and migraines was diagnosed with COVID-19 via a polymerase chain reaction test. Following diagnosis, he was initially managed in an outpatient setting with prednisone and azithromycin.

Second presentation

Nine days following his initial diagnosis, he presented to a critical access hospital following a syncopal episode. At this presentation, he was found to be hypoxic with SpO_2_ levels in the 70s on room air along with a blood glucose level of 466 mg/dL. Ketones were present in his urine. He was subsequently transferred to our tertiary care facility for intensive care unit (ICU) care secondary to respiratory failure and diabetic ketoacidosis.

On arrival to our ICU, initial laboratory studies demonstrated a lactic acid level of 4.2 mmol/L, C-reactive protein of 324.8 mg/L, and negative computed tomography angiography for pulmonary embolism. The patient did not require mechanical ventilation but required high-flow nasal cannula respiratory support with maximum oxygen flow at 60 L/minute with an FiO_2_ of 90%. He received tocilizumab, intravenous dexamethasone, azithromycin, and ceftriaxone throughout the course of his ICU admission. Lovenox was initiated for routine deep vein thrombosis prophylaxis. Five days after admission to the ICU, he was transferred out of the ICU and ultimately discharged home 10 days after his initial ICU admission. At discharge, his oxygen requirements were none at rest and 2-3 L with exertion. He was able to ambulate independently without any assistive devices.

Third presentation

**Figure 1:**
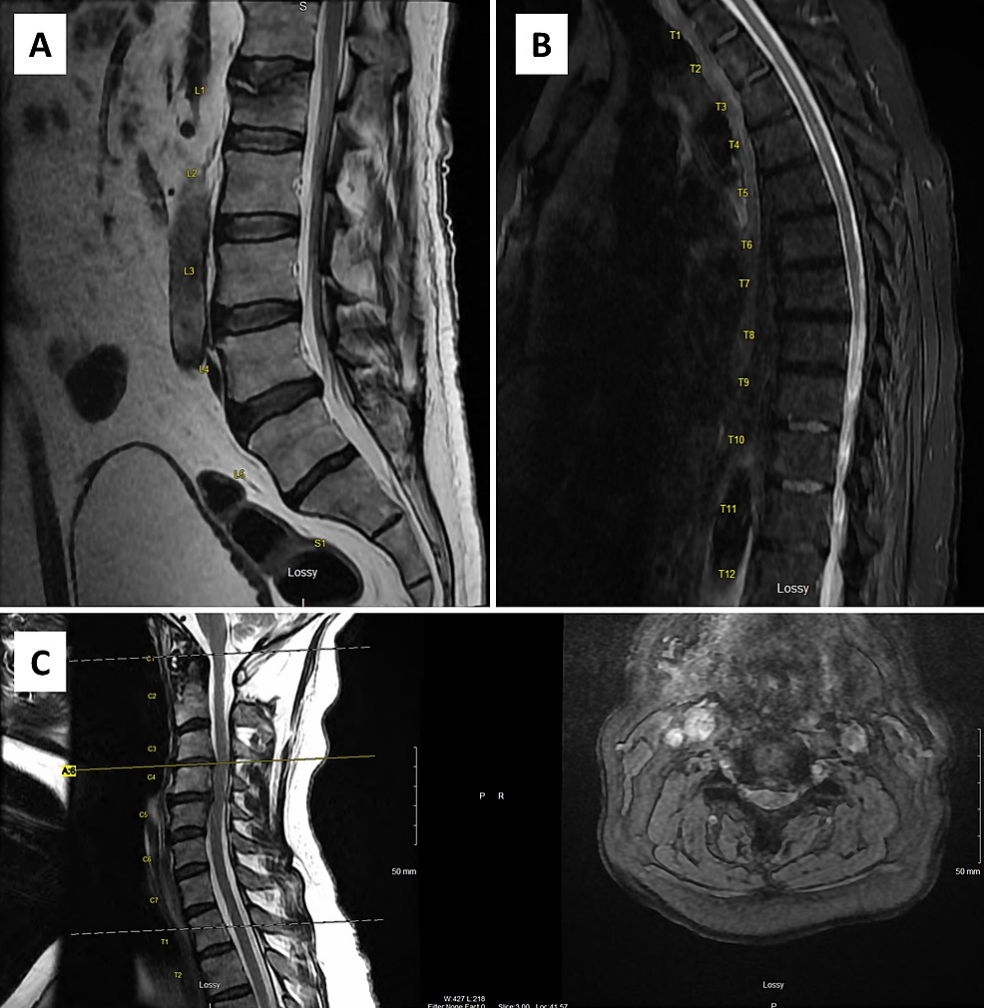
Magnetic resonance imaging of the lumbar and cervical spine. A: Sagittal T2 MRI image at the level of the midline lumbar spine. No radiographic evidence of central stenosis; however, there is significant distension of the bladder. B: Sagittal T2 MRI image at the level of the midline thoracic spine. No radiographic evidence of thoracic spinal cord compression. C: Left image illustrates sagittal T2 MRI image at the level of the cervical spine demonstrating significant central spinal cord compression with intraparenchymal T2 hyperintensity at the C3-4 and C4-5 vertebral levels. Panel C: Right image illustrates axial T2 MRI image at the level of the C3-4 disc. Severe compression of the spinal cord is demonstrated with corresponding intraparenchymal T2 hyperintensity. MRI: magnetic resonance imaging

Nine days after his discharge from the hospital, he returned to the emergency department with a six-day history of bilateral lower extremity weakness, low back pain, diminished sensation, and bowel and bladder incontinence. Examination revealed decreased rectal tone with absent rectal sensation. Workup for cauda equina syndrome was urgently pursued; however, magnetic resonance imaging (MRI) of the lumbar spine did not reveal compression of the cauda equina. An incidental, acute L1 superior endplate fracture with 30% loss of vertebral body height and minimal retropulsion was observed. Subsequent cervical and thoracic MRI was performed and revealed severe central stenosis at C3-4 and C4-5 with spinal cord flattening and intraparenchymal T2 hyperintensity at the corresponding levels (Figure 1).

A focus of reactive enhancement was noted in the T10 superior endplate without fracture or stenosis. Clinically, strength was 5/5 in the bilateral upper extremities. Strength was 4/5 in the bilateral proximal lower extremities and 4+/5 in the bilateral distal lower extremities. Reflexes were 2+ in the bilateral upper extremities and 1+ in the bilateral patellar and Achilles. There was a T12-L1 sensory level to light touch on examination with sparing of the plantar surface of his bilateral feet. Gait analysis revealed a maximal walking distance of 6 feet using a walker. Cerebrospinal fluid analysis revealed an elevated protein of 171 mg/dL (Table 1).

**Table 1:** Cerebrospinal fluid analysis. VDRL: venereal disease research laboratory; HSV 1: herpes simplex virus 1; HSV 2: herpes simplex virus 2; CMV: cytomegalovirus

	Value	Reference range
Appearance	Clear	--
White blood cell count	2 cells	0–8 cells
Glucose	64 mg/dL	40–70 mg/dL
Protein	171 mg/dL	15–60 mg/dL
Lymphocytes	63%	40–80%
Monocytes	37%	15–45%
Infectious Disease Panel		
VDRL	Negative	--
Cryptococcal	Negative	--
HSV 1	Negative	--
HSV 2	Negative	--
CMV	Negative	--

On hospital day two, he was started on Decadron 4 mg every eight hours. On hospital day three, he was started on a three-day course of intravenous immunoglobulin (IVIG) 500 mg/kg for suspected demyelinating polyradiculopathy. At this time, he was noted to have improved strength and reflexes in his bilateral lower extremities. Furthermore, he began to regain control of his bowels on hospital day three. On day six, his Foley catheter was discontinued and the patient demonstrated normal voiding habits without incontinence or retention. From a functional perspective, he was able to ambulate without assistance for 130 feet with breaks secondary to fatigue. He was ultimately discharged on hospital day seven with a five-week prednisone taper and follow-up appointments with neurosurgery and neurology. At the six-week outpatient neurosurgery follow-up, there was partial resolution of his motor symptoms, complete resolution of his bowel and bladder incontinence, and a GBS disability score of 3/6 [11]. Furthermore, there was an absence of frank signs of clinical myelopathy throughout the patient’s hospital course.

## Discussion

COVID-19 is a novel coronavirus that led to a global pandemic. While primarily a respiratory disease, recent evidence has suggested the potential neuroinvasive predilection of COVID-19. Although there are limited reports, neurologic symptoms identified secondary to a COVID-19 infection have included cranial nerve (1, 3, 7, 9, and 10) palsies, acute encephalopathy, acute necrotizing encephalopathy, and an acute immune-mediated polyradiculopathy consistent with GBS [3-5,9,12,13]. COVID-19 is thought to potentially cause GBS in a subset of patients through direct or indirect mechanisms. COVID-19 has a direct neuroinvasive capacity due to the presence of angiotensin-converting enzyme 2 receptors in neural tissues. Indirect mechanisms that have been postulated surround the significant inflammatory response that is mounted by the host in response to the viral antigens. Increased levels of interleukin-6 have been identified and stimulate the inflammatory cascade which is likely responsible for most neurological manifestations of COVID-19 infection [7,9,14]. Here, we present a patient who developed an acute demyelinating polyradiculopathy with rapid bowel and bladder involvement following a severe infection with COVID-19. Our patient’s presentation was initially pathognomonic for cauda equina syndrome; however, on further evaluation, no corresponding pathology was identified in the lumbar spine.

The main confounder in the rapid diagnosis of our patient’s case was the presence of severe central cervical spinal stenosis with spinal cord signal heterogeneity at C3-4 and C4-5. Despite the profound radiologic abnormalities, the patient did not demonstrate signs of myelopathy or an upper motor neuron injury. Following a close review of the symptom onset and the drastic positive response to treatment with IVIG and steroids, it was determined that our patient’s initial neurologic decline was a result of the prior COVID-19 infection and not his structural spinal abnormalities.

Our patient was ultimately diagnosed with an acute inflammatory demyelinating polyneuropathy or GBS that was preceded by typical signs and symptoms of biologically confirmed COVID-19 infection. The diagnosis of GBS in this patient was confirmed via cerebrospinal fluid analysis that revealed an albuminocytologic dissociation (Table 1) without other abnormalities. The development of GBS or acute inflammatory demyelinating polyneuropathy following a COVID-19 infection has become a well-known phenomenon with many small case reports discussing patients who demonstrated classical distal paresthesias with rapidly progressive limb weakness within the first five days of symptom onset [3,6]. Unique to our case, the patient developed rapid and severe decreased rectal tone along with bowel and bladder incontinence that has not been commonly reported or associated with post-COVID GBS [15,16]. Pourfridoni et al. recently speculated that COVID-19 inflammation and demyelination of the pudendal nerve could be a root cause of bladder and bowel incontinence; however, this has not been observed in the literature to date [17]. Importantly, our patient’s bowel and bladder function recovered rapidly following the initiation of steroids and IVIG therapy. The present report highlights the diverse neurological manifestations of COVID-19-associated GBS and the importance of a thorough neurological evaluation in these patients.

## Conclusions

We discuss the development of an acute inflammatory demyelinating polyneuropathy with predominant bowel and bladder involvement that was preceded by COVID-19 infection. Importantly, this patient’s case of COVID-19 was complicated by severe infection characterized by respiratory compromise requiring high-flow nasal cannula respiratory support. Given the novelty of the SARS-CoV-2 virus, the association between severe COVID-19 infections and neurologic consequences is not yet known. However, in this case, the confounding radiologic findings of severe central cervical spinal cord compression complicated the patient’s initial presentation. Following further diagnostic evaluation, an albuminocytologic dissociation on cerebrospinal fluid analysis was revealed and rapid resolution of symptoms following treatment with steroids and IVIG suggested a diagnosis of acute inflammatory demyelinating polyneuropathy. At subsequent follow-up visits, there was complete resolution of both his motor symptoms and bowel and bladder incontinence. Given the impact of the COVID-19 pandemic and the multitude of unknown consequences of the illness, further studies are crucial to understanding the effects of this infection on the nervous system in the short and long term. Therefore, surgeons and clinicians should carefully evaluate patients presenting with neurologic deficits and ensure a thorough examination to identify the appropriate etiology of a neurologic deficit to allow for prompt and effective intervention.

## References

[1] Caress JB, Castoro RJ, Simmons Z, Scelsa SN, Lewis RA, Ahlawat A, Narayanaswami P (2020). COVID-19-associated Guillain-Barré syndrome: the early pandemic experience. Muscle Nerve.

[2] Keddie S, Pakpoor J, Mousele C (2021). Epidemiological and cohort study finds no association between COVID-19 and Guillain-Barré syndrome. Brain.

[3] Paybast S, Gorji R, Mavandadi S (2020). Guillain-Barré syndrome as a neurological complication of novel COVID-19 infection: a case report and review of the literature. Neurologist.

[4] Koralnik IJ, Tyler KL (2020). COVID-19: a global threat to the nervous system. Ann Neurol.

[5] Galassi G, Marchioni A (2020). Facing acute neuromuscular diseases during COVID-19 pandemic: focus on Guillain-Barré syndrome. Acta Neurol Belg.

[6] Lascano AM, Epiney JB, Coen M (2020). SARS-CoV-2 and Guillain-Barré syndrome: AIDP variant with a favourable outcome. Eur J Neurol.

[7] Zhao H, Shen D, Zhou H, Liu J, Chen S (2020). Guillain-Barré syndrome associated with SARS-CoV-2 infection: causality or coincidence?. Lancet Neurol.

[8] Ellul MA, Benjamin L, Singh B (2020). Neurological associations of COVID-19. Lancet Neurol.

[9] Rahimi K (2020). Guillain-Barre syndrome during COVID-19 pandemic: an overview of the reports. Neurol Sci.

[10] Korem S, Gandhi H, Dayag DB (2020). Guillain-Barré syndrome associated with COVID-19 disease. BMJ Case Rep.

[11] van Koningsveld R, Steyerberg EW, Hughes RA, Swan AV, van Doorn PA, Jacobs BC (2007). A clinical prognostic scoring system for Guillain-Barré syndrome. Lancet Neurol.

[12] Trujillo Gittermann LM, Valenzuela Feris SN, von Oetinger Giacoman A (2020). Relation between COVID-19 and Guillain-Barré syndrome in adults. Systematic review. Neurologia (Engl Ed).

[13] Gale A, Sabaretnam S, Lewinsohn A (2020). Guillain-Barré syndrome and COVID-19: association or coincidence. BMJ Case Rep.

[14] Zhou Z, Kang H, Li S, Zhao X (2020). Understanding the neurotropic characteristics of SARS-CoV-2: from neurological manifestations of COVID-19 to potential neurotropic mechanisms. J Neurol.

[15] El Otmani H, El Moutawakil B, Rafai MA (2020). Covid-19 and Guillain-Barré syndrome: more than a coincidence!. Rev Neurol (Paris).

[16] Camdessanche JP, Morel J, Pozzetto B, Paul S, Tholance Y, Botelho-Nevers E (2020). COVID-19 may induce Guillain-Barré syndrome. Rev Neurol (Paris).

[17] Pourfridoni M, Pajokh M, Seyedi F (2021). Bladder and bowel incontinence in COVID-19. J Med Virol.

